# Baleen hormone analyses reveal stress and reproductive life-history of the critically endangered Rice’s whale (*Balaenoptera ricei*)

**DOI:** 10.1371/journal.pone.0347749

**Published:** 2026-05-13

**Authors:** Rebecca G. Evey, Matthew S. Savoca, John Ososky, Michael R. McGowen, Jeremy A. Goldbogen, Michael Cherney, Allison Case, Kathleen E. Hunt

**Affiliations:** 1 Department of Biology, George Mason University, Manassas, Virginia, United States of America; 2 Hopkins Marine Station, Stanford University, Pacific Grove, California, United States of America; 3 Department of Vertebrate Zoology, Smithsonian National Museum of Natural History, Washington D.C., United States of America; 4 University of Michigan Medical School, Ann Arbor, Michigan, United States of America; 5 Marine Mammal Institute, Oregon State University, Newport, Oregon, United States of America; 6 Smithsonian-Mason School of Conservation, Front Royal, Virginia, United States of America; Natural History Museum of London, UNITED KINGDOM OF GREAT BRITAIN AND NORTHERN IRELAND

## Abstract

The Rice’s whale (*Balaenoptera ricei*) is a recently described species of baleen whale found in the Gulf of Mexico. With fewer than 50 adults remaining, the Rice’s whale is the most endangered baleen whale species. Analyses of reproductive and adrenal hormones promote the understanding of reproductive cycles and stress physiology in this rare and poorly understood species. Baleen plates contain steroid hormones stored throughout the period of baleen growth and have been used for continuous, multi-year retrospective assessment of the reproductive and glucocorticoid history of individual whales. We measured progesterone, testosterone, cortisol, and corticosterone in baleen plates of seven individual Rice’s whales (four males — one of which was the holotype — and three females), including two individuals believed to have died from starvation and one known to have been killed by ship strike. Baleen powder was obtained by drilling every 1 cm (~15–30 day intervals) from the base of the plates to the distal end. Hormones were quantified with enzyme immunoassay kits, and presence of the specific hormones was further confirmed with analytical chemistry. All assays passed validation assays for Rice’s whale baleen extract. In the two individuals that likely died of starvation, all four steroid hormones show increases in the most recently grown baleen, a pattern observed in other baleen whales in cases of prolonged illness or injury before death. A female with a known recent pregnancy had a sustained elevation of progesterone spanning the majority of her plate, indicating that baleen analysis in this species can detect recent pregnancies. No evidence of annual testosterone cycles was noted in three adult males, suggesting that this subtropical species might not have strong seasonal reproduction, which is atypical for baleen whales. Thus, we conclude that baleen hormone analysis can be used to clarify life history patterns in this critically endangered species.

## Introduction

The Rice’s whale (*Balaenoptera ricei*) is a recently identified species of whale thought to reside year-round within the Gulf of Mexico (GoM, also known as “Gulf of America” within the USA). This species was once considered a distinct population of the Bryde’s whale (*Balaenoptera edeni*), but in early 2021, genetic and morphological analyses confirmed it is a distinct species [1,2]. The Rice’s whale is critically endangered and is the most endangered baleen (mysticete) whale species, with the total population size estimated at 50–100 individuals, and fewer than 50 mature adults [2–5]. Unlike many mysticetes that migrate seasonally between temperate or polar feeding grounds in the summer and tropical breeding grounds in the winter, Rice’s whales are believed to be non-migratory and appear to mainly reside in the northeastern GoM, with additional sightings and acoustic detections stretching across the northwestern GoM [1,5–11], although its historic range may once have been broader. Acoustic evidence indicates that Rice’s whales occur in Mexican waters as well [11]. Due to its extremely small population size, newly elevated taxonomic status, and numerous anthropogenic threats in the GoM, conservation efforts are urgently needed to protect this critically endangered species [12]. However, knowledge gaps pose roadblocks for developing an effective recovery plan. For example, more information is needed on reproductive timing and seasonality, as well as the potential physiological effects of anthropogenic stressors such as ship traffic, entanglement in fishing gear, and exposure to pollutants and debris.

Hormone quantification can be a powerful tool to reveal physiological states of an animal that may not be detectable otherwise. Analyses of reproductive hormones and adrenal glucocorticoid (GC) hormones are useful for conservation and management, particularly analyses of patterns over time [13–16]. However, it can be difficult to obtain the necessary samples from rare cetaceans. Recently, baleen analysis has become increasingly employed for such hormone analyses in cetaceans [17–25]. Baleen is a keratinized feeding tissue found in all extant mysticete species, consisting of multiple baleen “plates” (vertical strips) that form a filter-feeding apparatus. Each plate grows slowly and continuously from well-vascularized gum tissue in the upper palate known as the ‘*Zwischensubstanz*’ and slowly degrades or frays at the distal tip [26–29]. Multiple studies in other mysticete species demonstrate that patterns of hormone concentrations within a baleen plate correspond to known events (e.g., pregnancy or entanglement) that occurred while that section of baleen was grown, implying that steroid hormones in circulating blood are incorporated in baleen as it grows [20–24,30,31]. Therefore, a baleen plate is thought to contain multiple, continuous years of hormone data, with the concentration of hormones at each point along the plate representing a certain point in time when that region of baleen grew.

From a conservation perspective, it is important to know the breeding season of any endangered species to assess whether certain management policies may be required during a certain season or region. It is currently unknown whether Rice’s whales are seasonal breeders, and, if so, what their breeding season may be. Some populations of the closely related Bryde’s whale can breed year-round with no seasonal restriction, but breeding peaks can be seen in autumn, and at least one Bryde’s population breeds seasonally in winter [32]. The occurrence and timing of seasonal reproduction in vertebrates can often be estimated via inspection of temporal patterns in reproductive hormones, particularly testosterone in males (e.g., [33–37]). In most seasonally breeding mammals, circulating plasma testosterone concentrations of adult males begin rising approximately a month prior to breeding (corresponding with initiation of spermatogenesis) and remain elevated throughout the breeding season [38]. Adult males of several mysticetes that breed seasonally show distinct elevations of testosterone in baleen grown during the putative breeding season (e.g., bowhead whale *Balaena mysticetus*, North Atlantic right whale *Eubalaena glacialis*, and blue whale *Balaenoptera musculus*) [39,40]. Thus, examining patterns of testosterone in Rice’s whale baleen could help illuminate whether this species breeds seasonally.

Progesterone is a key mammalian reproductive hormone that is responsible for maintaining the uterine endometrium in pregnant females, and thus it is typically significantly elevated for a prolonged period during pregnancies, often reaching a peak in the second half of gestation and declining at parturition. Thus, progesterone can be used as a biomarker for mammalian pregnancy confirmation as well as (in some species) for identifying the stage of pregnancy [41,42]. In multiple mysticete species, sustained elevations of progesterone in baleen correspond to documented periods of pregnancy (i.e., confirmed from calf sightings), for example, in bowhead, North Atlantic right whale, and humpback whales [17,24,31]. In those species with long baleen grown across >3 years, it is often possible to confirm prior pregnancies, estimate gestation length and inter-calving intervals and sometimes estimate age of sexual maturity (i.e., from patterns of progesterone elevations across the baleen). However, Rice’s whale baleen plates are relatively short, capturing only an estimated one to two years of hormone data. Though examination of inter-calving interval may not be possible with such short baleen, pregnancy confirmation and estimation of gestation length might still be possible. Thus, testosterone patterns of males and progesterone patterns of females could shed light on 1) breeding seasonality, 2) pregnancy determination, and 3) gestation length.

Glucocorticoids (GCs), too, can provide useful information for conservation and management. In vertebrates, exposure to unusual or prolonged stressors stimulates the hypothalamic-pituitary axis (HPA axis), resulting in elevation of the GCs, typically cortisol and/or corticosterone, which together coordinate an adaptive stress response that helps reallocate energy from non-essential functions (digestion, growth, reproduction, etc.) to functions essential for coping with the stressor (muscle activity, oxygen transport, etc.) [43,44]. Consequently, periods of prolonged stress with consistently elevated GCs can have negative impacts on health, reproduction, and survival. Rice’s whales are likely exposed to numerous anthropogenic stressors in GoM waters, including vessel strikes, fishing gear entanglement, anthropogenic noise, habitat destruction, oil spills, and fishery interactions [4,12]. The relative impacts of these stressors on this species are unknown, and thus, determination of typical patterns in GCs across time, and comparison of GC patterns after certain types of stressors (e.g., acute vs. chronic causes of death), could prove fruitful. Analysis of both GCs (i.e., rather than just one) can produce additional information, as cortisol and corticosterone often show different patterns that appear to vary with type and duration of the stressor [19,20,22,39,45].

Accurate determination of the timeline represented by a given baleen plate (e.g., number of years or months represented by a single plate) requires information about baleen growth rate (BGR), measured in cm of length along the primary growth axis added per year, for that species and age class. In seasonally migrating mysticetes, a timeline estimation is typically achieved through analysis of predictable annual cycles in stable isotopes corresponding with known migration patterns and related seasonal shifts in prey [31,46,47]. However, since it is believed Rice’s whales do not migrate, this approach may not be viable. Fortunately, BGR appears relatively conserved across mysticetes, with age class generally having a greater effect than species (e.g., adult BGR often ~14–16 cm/yr, and juveniles BGR > 20 cm/yr) [48]. In this study, we use BGR of the closely related Bryde’s whale (14 cm/yr for adults) to estimate BGR for Rice’s whales [48].

This study’s goal is to close knowledge gaps regarding reproduction and stress in the Rice’s whale through retrospective, time-series analyses of hormones obtained from baleen, specifically testosterone, progesterone, cortisol, and corticosterone. We analyzed patterns of these four hormones in the baleen of seven different Rice’s whale individuals to address the following objectives: (1) Determine whether steroid hormones can be accurately quantified in Rice’s whale baleen with commercial immunoassay kits. (2) Assess patterns of testosterone over time in three adult males to examine occurrence of any seasonal peaks indicative of predictable breeding season, with the tentative hypothesis that all three males would show elevated testosterone at the same month(s) of the year. (3) Determine whether pregnancy can be diagnosed in this species from patterns in baleen progesterone of females, with the hypothesis that in one case of a female known to be lactating at death, progesterone would be highly elevated in baleen grown the year prior. (4) Examine whether patterns of GCs at the base of the plate (most recently grown baleen) correlate with known cause of death (e.g., acute vs chronic), with the hypothesis that GCs would be elevated in two cases of known chronic stress prior to death, as compared to a case of known acute cause of death. (5) Determine typical concentrations and ranges of hormones in two subadults and a calf; no specific hypotheses were framed for these cases because little endocrine information exists for these age classes in mysticetes. Though our sample size is small, it represents the first endocrine information from this species. Thus, examining these seven case studies may illuminate some basic aspects of reproductive and stress biology for this critically endangered species, to help set research priorities and inform potential management actions.

## Materials and methods

### Baleen samples

Seven individual whales are included in this study (Table 1); one baleen plate from each individual was obtained from the Smithsonian National Museum of Natural History (USNM). These included three adult males (USNM 572992, USNM 504074, USNM 594665 [the holotype for this species]); one adult female (USNM 593536); one subadult male (USNM 239307); one subadult female (USNM 504768); and one female calf (USNM 593537). These plates were collected over a century, from 1923 (USNM 239307) to 2019 (USNM 594665), with two individuals collected in the 1970s (USNM 504074 and 504768), and the rest from the early 21^st^ century (Table 1). These seven specimens represent all available Rice’s whale baleen at the time of the study. All baleen plates except one had intact bases, meaning the baleen at the base of the plate should correspond approximately with the date of death. USNM 504768 was the only baleen plate missing the base (i.e., baleen plate was obtained at necropsy by means of cutting the plate at the gumline, thus removing the embedded base of the plate), and consequently the timeline of this baleen plate cannot be anchored to a specific date (though approximate dates — i.e., the number of months represented by the remaining portion of the plate — can be estimated). Another specimen, USNM 504074, is believed to have an intact or near-intact base but may have been missing ~1 cm of the proximal-most portion of the base (based on visual comparison with other specimens). Three juvenile whales were included in the study (USNM 239307 (male), USNM 504768 (female), USNM 593537 (female)), two of which were tentatively classed as “subadults” (i.e., larger than calves and likely post-weaning but not yet adult) based on body length between 50–90% of adult length (Table 1). The distal portions of these two individuals’ plates may reflect hormone concentrations during their first year of life when they were likely nursing. The remaining individual was classed as a “calf” (estimated age < 1 year, likely nursing) based on a total body length of 4.7 meters (i.e., < 50% of adult length). Isotope and contaminant analyses of these same seven specimens will occur in future studies.

**Table 1:** Known information on the Rice’s whale individuals of this study.

Whale USNM^1^ ID	Age	Sex	Length (m)	Date Collected	Location	Death	Additional Information	Subsamples Attained
594665	Adult	M	11.26	28-Jan-2019	Flamingo, FL	Plastic ingestion, GI perforation starvation	Holotype	20
572992	Adult	M	11.05	12-Mar-2003	New Hanover, NC	Entanglement resulting in starvation	--	22
504074	Adult	M	19	30-May-1974	Tarpon Springs, FL	Unknown	--	23
593536	Adult	F	12.65	4-Oct-2009	Hillsborough, FL	Ship strike	Lactating at point of death	21
239307	Subadult	M	8.01	18-Mar-1923	Walnut Point, VA	Unknown	--	18
504768	Subadult	F	8.69	14-Mar-1978	Duval, FL	Unknown	--	12
593537	Calf	F	4.7	2-Nov-2006	Sandestin, FL	Unknown	Possible weaning at point of death	5

^1^USNM ID = Catalog number from the Smithsonian National Museum of Natural History.

### Baleen subsampling

Baleen plates were triple rinsed with 95% ethanol to remove surface contamination, allowed to dry, and subsampled sequentially and along the primary axis of growth every 1 cm (with a few samples at an increment of 1.5 cm apart due to damage on the baleen) beginning at the dorsal (proximal) buccal edge, using an electric rotary grinder (Dremel model 4000) with a 1.25 cm sanding band attachment to grind baleen into a fine powder. The grinder tip was also triple rinsed with 95% ethanol between each sample. Overall, 121 discrete baleen samples were collected across 7 baleen plates from 7 different individuals. As each plate was subsampled at regularly spaced intervals, sample size varies for each individual according to the length of the plate. Total number of samples per individual ranged from 5 samples (calf) to 23 samples (adult); see Table 1 for details.

### Hormone extraction

From each sample of baleen powder, a 20 ± 0.9 mg subsample was weighed on a scale with 0.1 mg repeatability (Sartorius Entris II Essential Analytical Balance) using an ionizer to decrease the effects of static charge on apparent sample mass (Ohaus ION-100A). The weighed baleen powder was then mixed with 4.00 mL of 100% HPLC-grade methanol in 16x100 mm borosilicate glass tubes, capped, shaken for 2 hours on a rack vortexer set at 40 motor speed (Glas-Col Large Capacity Mixer), then centrifuged for 15 minutes at 4000 g (Thermo Scientific Sorvall ST4R Plus Series). After centrifugation, 3.50 ml of methanol supernatant (containing hormones) per sample was pipetted to a 13x100 mm borosilicate glass tube, with pellets discarded. Only 3.50 mL of the 4.00 mL supernatant was recovered to avoid pipettor disruption of the pellet; final data were corrected accordingly. The supernatant was then dried down in a rotary evaporator under vacuum at 45°C (Thermo Scientific Savant SpeedVac SPD1030 Integrated Vacuum Concentrator) until dry. Dried samples were reconstituted in 500 ul of assay buffer (“X065” buffer, Arbor Assays, Ann Arbor, MI), hand vortexed for 10 seconds (Vortex-Genie 2 Digital), shaken for 1 minute on the rack vortexer, and then placed in a water-bath sonicator for 5 minutes (Branson 3800 Ultrasonic Cleaner). The resulting samples are considered the full-strength (“1:1,” “neat”) sample. Samples were then transferred to o-ring-capped cryovial tubes and stored at −80°C until assay. All samples were assayed within 1 month of extraction.

### Validation assays

Validations, including parallelism and accuracy, were conducted using pooled samples from Rice’s whale baleen powder tested with commercial enzyme immunoassay kits (progesterone kit #K025, testosterone kit #K032, cortisol kit #K003, and corticosterone kit #K014, Arbor Assays, Ann Arbor, MI). The sample pool was created from two adult males (USNM 594665 and USNM 572992) due to the excess amount of sample mass available.

Parallelism validation, which evaluates binding affinity of the assay antibody to sample hormone, was tested by creating a serial dilution of the pool in assay buffer and assaying all dilutions alongside known-dose standards for progesterone, testosterone, cortisol, and corticosterone. Results for both the serial dilutions of the pool and standards were plotted as the percentage bound vs. the logarithm of the relative dose. The slopes of the linear portion of the curves were compared for parallelism, which would indicate similar binding affinity of assay antibody to sample hormone as compared to pure hormone, and is considered strong, but not definitive, evidence that the sample contains the hormone of interest (or a closely similar metabolite). The presence of specific hormones of interest was further confirmed via high-performance liquid chromatography-mass spectrometry (LC-MS/MS) of selected samples (see below).

Accuracy (aka “matrix effect test”) was next tested by analyzing the slope of the expected standard dose vs. standards spiked with diluted pool (i.e., the dilution selected for routine assay), to determine the assay’s accuracy for discriminating low from high doses in the presence of sample matrix at a given dilution. Accuracy validations for all four hormones involved assays of a set of standards spiked with 50 μl pooled baleen extract (1:4 for progesterone and testosterone, 1:1 for cortisol and corticosterone) and a set of standards spiked only with 50 μl assay buffer. Results were graphed as apparent dose of standards spiked with pool vs. known standard dose, with acceptable accuracy defined as a straight line with a slope close to 1.0 (see Statistical Analysis for detailed criteria).

### Hormone assays

After successful validations (see Results), all samples were then analyzed for immunoreactive progesterone, testosterone, cortisol, and corticosterone using enzyme immunoassays. Samples were assayed at 1:1 for cortisol and corticosterone but were diluted to 1:4 for progesterone and testosterone assays (except for USNM 593536 diluted to 1:40 for progesterone, due to very high concentrations of progesterone) to keep results as near as possible to the linear portion of the standard curve (for greatest assay precision). Each whale’s samples were analyzed within the same assay, and the order of the samples was pseudo-randomized to minimize influences of intra-assay variation on longitudinal data. The manufacturer’s assay protocols were used, but with one additional low-dose standard added by extending the standard curve for one more serial dilution. The cortisol assay was run using X065 buffer (rather than the X053 buffer indicated by the protocol) based on the manufacturer’s technical advice; this change was performed to keep all assays in the same buffer, which minimizes the loss of sample volume due to pipetting. Assays included quadruplicates of nonspecific binding wells and blanks (zero doses), and duplicates of standards, samples, and controls. Any standard with a coefficient of variation (CV) >10% between wells was omitted from the standard curve. Any sample with a CV > 10% between wells was re-assayed, with the exception of one sample (USNM 594665 subsample 2 (1 cm), 20.3% CV) that did not have sufficient extract for re-assay. All assay results were converted to nanograms of immunoreactive hormone per gram of baleen powder.

### Mass spectrometry

To confirm the chemical identity of immunoreactive hormones detected by assay antibodies, four samples of Rice’s whale baleen powder were selected for mass spectrometry analysis. Four samples were sufficient to verify that the putative hormones detected by immunoassay antibodies are in fact present in baleen extracts from this species. Two samples were selected from the pregnant female (i.e., the only adult female in the study, with the two samples representing different stages of pregnancy), one from an adult male, and the fourth from a female calf (representing a non-adult).

To extract steroid analytes from powder samples, we adapted the procedure documented in [49] for dentin powder samples. We loaded each sample with an extraction solution of 1.5 ml HPLC-grade methanol (Fisher Scientific) and 100 µl internal standard made from deuterated forms of each analyte in a 40% methanol solution, along with ~250 mg Lysing Matrix D beads (MP Biomedicals) into a 2-ml screw-cap centrifuge tube (MP Biomedicals). The beads prevented clumping and kept powder samples in suspension during agitation and rotation. After adding the solution, each tube was vortexed for 60 s at 10,000 rpm and rotated at 30 rpm for 3 h at room temperature. Samples were then centrifuged at 12,700 rpm for 8 min, and supernatant (1450 µl) was transferred to a 2-ml autosampler vial and evaporated to dryness in a vacuum concentrator (Savant SpeedVac SPD1030) for 2 h without heating and using a pre-cooled rotor. Evaporated samples were kept on ice until reconstituted with 40 µl HPLC-grade methanol and vortexed at 10,000 rpm for 20 s. After reconstitution, samples were diluted with 300 µl HPLC-grade water (Fisher Scientific) and vortexed for an additional 20 s. We then performed supported liquid extraction (SLE) using preloaded 400 µl cartridges (Biotage Isolute SLE+), eluting with 1.8 ml HPLC-grade methyl *tert*-butyl ether (Honeywell) that was subsequently evaporated to dryness under vacuum for 25 min without heat in a pre-cooled rotor. Samples were kept on ice until being reconstituted again, this time with 200 µl 40% methanol, vortexing at 10,000 rpm for 20 s. Reconstituted samples were stored at –20 °C overnight for analysis the subsequent day.

Steroid analyses were performed on an Agilent 6490 triple quadrupole mass spectrometer and Agilent 1260 and 1290 dual front-end HPLC/UPLC using a previously published method [50]. Multiple controls (processed methanol without sample, processed water, an internal pooled serum control, and an internal quality control mixed from stock steroids) were included in the sample run, and a calibration curve was calculated using seven calibration standards with concentrations spanning the range detected in samples. Concentrations of analytes (based on a calculation that assumed specimen sample size of 100 mg) were calculated based on comparison to the known concentrations of deuterated standards added to the extraction solution. Steroids were measured using multiple reaction monitoring (MRM). In MRM, two of the ions produced by controlled destruction of the precursor ion (the charged steroid molecule) are selected to be the quantifier and qualifier ions. The integration of the quantifier ion peaks of each steroid and deuterated internal standard are used to calculate steroid concentration, while the qualifier ion confirms the identification of the quantifier ion. Results were considered unreliable when ratios of qualifier and quantifier ion were outside of expected, empirically determined ranges. Final steroid levels in baleen samples were calculated by converting measured concentrations according to sample mass.

### Statistical analysis

Parallelism assays were analyzed using F-tests on the linear portions of the curves, with a nonsignificant difference in slopes (i.e., *p* > 0.05) indicating acceptable parallelism. Accuracy assays inspected for goodness-of-fit of the linear regression line as well as slope, with *r*^2^ > 0.90 and a slope between 0.7–1.3 indicating acceptable accuracy [17].

Descriptive statistics for each hormone included calculation of range and baseline. For each whale, the baseline ng/g of each hormone was calculated using an iterative process of removing data points more than two standard deviations higher than the mean until no outlying points remained [51]. The resulting mean then indicates the baseline of that hormone for that whale. Peaks were then identified as any point greater than two standard deviations above that baseline [51,52]. Male testosterone peaks, female progesterone peaks, and GC peaks in both sexes were then visually evaluated for correspondence with estimated time of year (testosterone peaks in males), relationship to documented pregnancy (for the lactating female), and/or co-occurrence with any documented causes of death (from necropsy reports). For inspection of potential testosterone seasonality in males, points along the three male baleen plates were assigned an estimated month of growth (albeit in different calendar years; see Table 1) using the known month of death and the estimated baleen growth rate. The three males’ testosterone profiles were then aligned by month to inspect any apparent synchronization in testosterone patterns indicating potential seasonality (Fig. 4). Additionally, each male’s testosterone profile was individually inspected for evidence of annual cyclicity (i.e., successive testosterone peaks occurring in the same month across different years). (Note that the shortness of Rice’s whale baleen and the proxy BGR derived from Bryde’s whale baleen limits this approach to an expected two successive annual testosterone cycles per baleen plate.) Finally, duration of gestation was estimated based on the timing of the sustained elevation of progesterone, patterns of GCs suggestive of parturition, and Bryde’s whale BGR.

Data were analyzed using GraphPad Prism version 10.2.0 (parallelism and accuracy) and R packages dplyr, ggplot2, and hormlong [52–55].

## Results

### Validations

**Fig 1:**
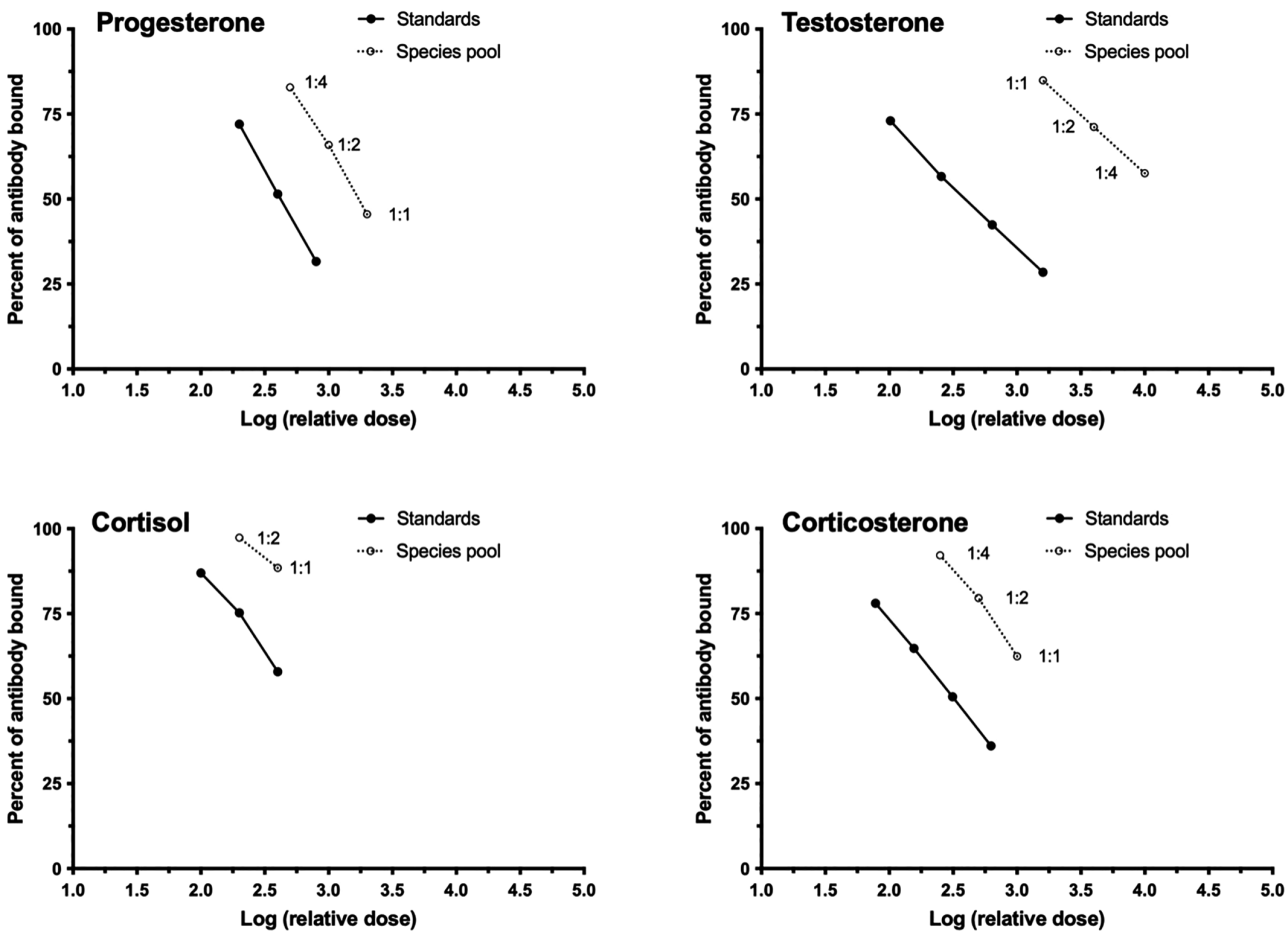
Assay parallelism curves. Parallelism assay results for validation of Rice’s whale baleen pooled extracts for progesterone, testosterone, cortisol, and corticosterone. Parallel slopes of standards and species pool indicate acceptable parallelism.

**Fig 2:**
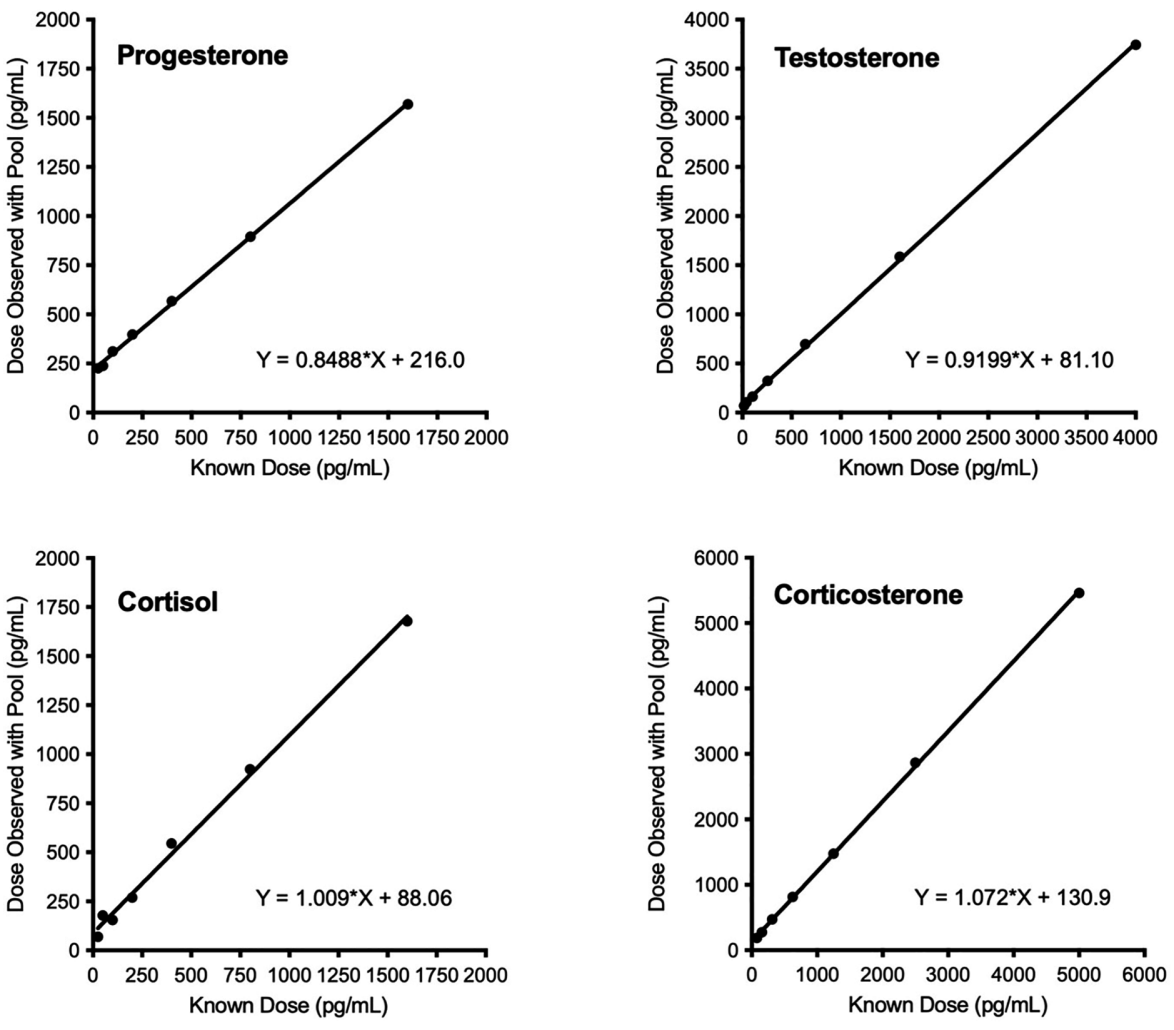
Assay accuracy linear regressions. Accuracy assay results for validation of Rice’s whale baleen pooled extracts for progesterone, testosterone, cortisol, and corticosterone. Slopes close to 1.0 demonstrate good accuracy.

All assays demonstrated robust parallelism and accuracy for Rice’s whale baleen extracts. The parallelism assays yielded slopes of serially diluted sample that were not significantly different from slopes of hormone standards (progesterone pool, *F*_1,5_ = 2.293, *P* = 0.2692; testosterone pool, *F*_1,5_ = 3.242, *P* = 0.1696; cortisol pool, *F*_1,5_ = 2.413, *P* = 0.3641; corticosterone pool, *F*_1,5_ = 0.7938, *P* = 0.4386). All assays also demonstrated acceptable accuracy (progesterone, *r*^2^ = 0.9993, slope = 0.8488; testosterone, *r*^2^ = 0.9997, slope = 0.9199; cortisol, *r*^2^ = 0.9954, slope = 1.009; corticosterone, *r*^2^ = 0.9998, slope = 1.072) (Figs 1 and 2).

Mass spectrometry results confirmed that Rice’s whale baleen powder contains native progesterone, testosterone, cortisol and corticosterone (i.e., the four hormones quantified by immunoassay), as well as multiple other steroid hormones (16-OH-progesterone, 17-OH-progesterone, androstenedione, 18-OH-corticosterone, 11-deoxy-cortisol, 18-OH-cortisol, aldosterone and estrone). Progesterone was the dominant progestogen detected (i.e., concentrations higher than other progestogens in all four samples tested). For androgens, however, androstenedione was unexpectedly found at a higher concentration than testosterone in three of four samples (all but the calf sample); that said, testosterone was also readily detectable in all four samples. Corticosterone was the dominant GC detected in all four samples, in agreement with assay results (Table 2). These mass spectrometry results confirm that Rice’s whale baleen contains the hormones targeted in the EIAs.

**Table 2:** Descriptive statistics for enzyme immunoassay results of Rice’s whale baleen samples. For full data see supporting information (S1 Table).

	Whale USNM ID	Progesterone		Testosterone		Corticosterone		Cortisol	
	Baseline (ng/g)	Range (ng/g)	Baseline (ng/g)	Range (ng/g)	Baseline (ng/g)	Range (ng/g)	Baseline (ng/g)	Range (ng/g)
Adult Males	594665	14.53	12.50-42.04	8.68	6.73-23.18	1.815	1.09-7.95	1.019	0.29-3.87
	572992	16.83	9.06-24.20	7.01	2.64-8.72	1.968	1.32-4.12	0.663	0.08-2.03
	504074	35.51	22.87-62.32	15.17	10.23-26.14	4.275	3.05-10.09	1.427	0.55-4.36
	Combined	22.29	9.06-62.32	10.32	2.64-26.14	2.69	1.09-10.09	1.04	0.08-4.36
Adult Female	593536	--^1^	44.78-1181.29	8.47	5.60-36.27	4.767	2.84-41.74	0.88	0.00-4.62
Subadults/Calf	239307	44.42	30.63-269.82	18.94	12.48-33.15	5.55	3.01-22.08	3.07	1.96-33.15
	504768	68.52	59.75-75.56	48.4	34.39-72.63	8.23	5.53-11.48	5.34	4.26-7.52
	593537	37.84	21.87-58.06	24.44	17.18-32.46	5.41	3.49-7.20	3.15	1.99-4.49
	Combined	50.26	21.87-269.82	30.59	12.48-72.36	6.4	3.01-22.08	3.85	1.96-33.15

^1^ No baseline could be calculated for the adult female due to a prior pregnancy influencing most data points.

### Adult males

**Fig 3:**
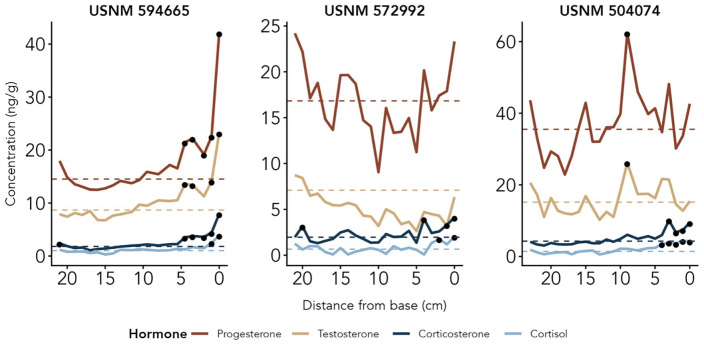
Adult male Rice’s whale hormone profiles.

**Fig 4:**
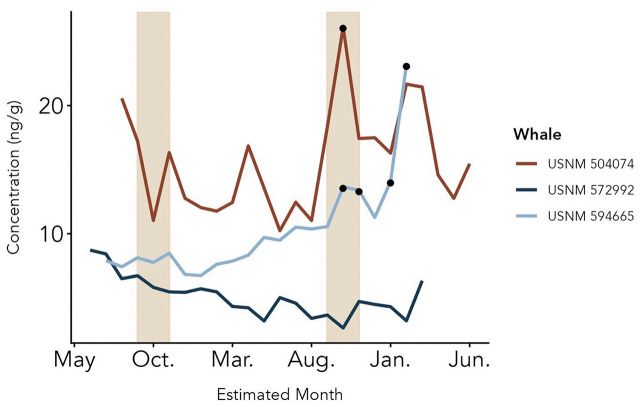
Adult male Rice’s whale testosterone patterns aligned by time of year.

In all three adult males (USNM 594665, USNM 572992, USNM 504074), the progesterone range and average baseline was higher than the testosterone range and average baseline (Figs 3 and 4; Table 2). The progesterone concentration in males were still five times less than the adult female’s progesterone levels during the non-pregnancy period. Corticosterone concentrations were consistently higher than cortisol concentrations (Fig 3; Table 2). All three males show peaks in GCs at the base of the plate, indicating a rise in GC circulation during the month(s) immediately prior to death (Fig 3; Table 2). Peaks in testosterone and progesterone at the base of the plate were also seen in male USNM 594665 (0–4.5 cm) corresponding to the GC peaks (Fig 3). Testosterone patterns across the three males did not show synchronized peaks in the same months, and none of the three males demonstrated any apparent cyclicity across the baleen plate (Fig 4).

Hormone profiles of three adult male Rice’s whales (USNM 594665, 572992, and 504074) from observed immunoreactive hormone concentrations across the baleen plates. Note the differing y-axes. Hormone concentrations (ng/g) are plotted by distance from base (0 being the base, i.e., the point grown most recently before death) to read from oldest (left) to newest (right) baleen growth. Dashed horizontal lines represent baseline and black points represent peaks, defined as values two standard deviations or more above baseline.

Testosterone profiles of three adult male Rice’s whales (USNM 504074, 572992, and 594665) from observed immunoreactive hormone concentrations across the baleen plates, aligned by estimated month of growth of each sample of baleen. Hormone concentrations (ng/g) are plotted by estimated month of baleen growth determined by the death date of each whale and average baleen growth rate. Shaded areas indicate typical breeding season of closely related Bryde’s whale. Black points represent peaks, defined as values two standard deviations or more above baseline.

### Adult female

**Fig 5:**
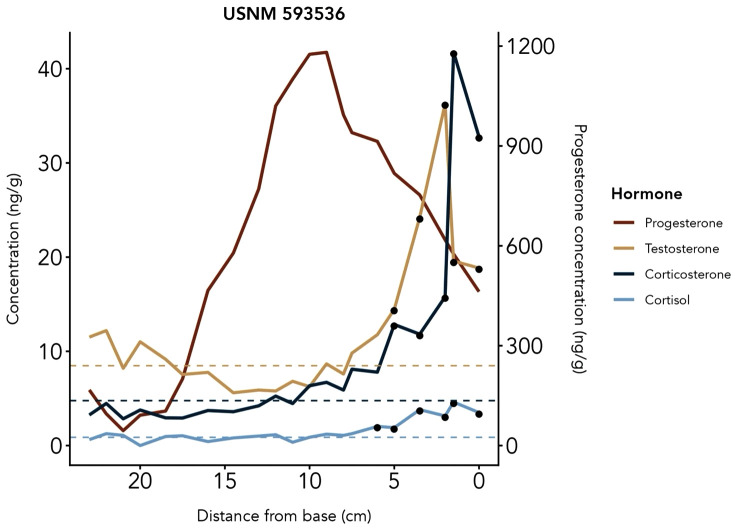
Adult female Rice’s whale hormone profile.

USNM 593536, the sole adult female in the study, was lactating at the time of death. A sustained elevation of progesterone was detected across most of the baleen plate, with a decline in progesterone starting near the middle of the plate continuing to the base of the plate (proximal end), interpreted as a pregnancy followed by a parturition event. Due to this individual’s large range in progesterone concentrations (Table 2), a baseline could not be calculated. Progesterone began to rise at 17.5 cm (measured from 0 cm = proximal-most end, i.e., most recently grown baleen), peaked at 9 cm at 1181.29 ng/g, followed by a gradual decline (Fig 5). The female’s progesterone average was 34.5 times greater than the average baseline of the three adult males, while her testosterone baseline was comparable to those of the males (Table 2). Corticosterone concentrations were consistently higher than cortisol, as seen in samples from adult males.

The proximal-most portion of the female’s baleen plate contained several near-simultaneous patterns of interest: a rise in testosterone occurs between 0–5 cm, peaking at 36.27 ng/g at 2 cm; and both GCs peaked simultaneously at 1.5 cm (with maxima of 4.62 ng/g of cortisol, and 41.74 ng/g of corticosterone). Based on a tentative beginning of gestation at 17.5 cm and a tentative parturition at 1.5 cm, this pregnancy spanned an estimated 16 cm. Using the Bryde’s whale BGR of 14 cm/year, the gestation duration of this individual was estimated as 13.7 months.

Hormone profile of the adult female Rice’s whale USNM 593536 from observed immunoreactive hormone concentrations across the baleen plate. Hormone concentrations (ng/g) are plotted by distance from base (0 being the base, i.e., the tissue grown most recently before death) to read from oldest (left) to newest (right) baleen growth. Dashed horizontal lines represent baseline and black points represent peaks, defined as values two standard deviations or more above baseline. Testosterone, corticosterone, and cortisol are plotted on the right y-axis and progesterone is plotted on the left y-axis.

### Subadults and Calf

**Fig 6:**
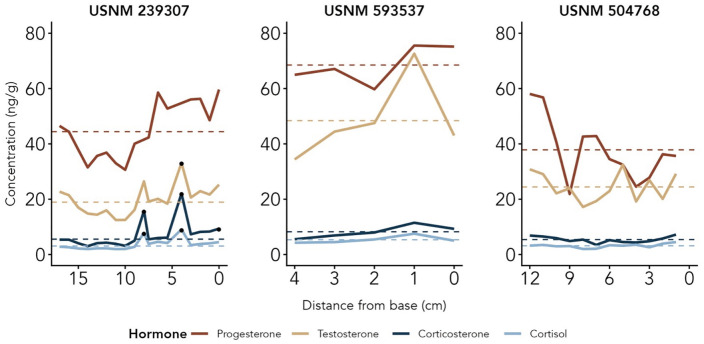
Juvenile Rice’s whale hormone profiles.

Unexpectedly, all three juveniles — both subadults and the calf — had higher testosterone, progesterone, and GC baselines than the four adults, with progesterone baselines higher than testosterone for all three regardless of sex (Fig 6 and Table 2).

Hormone profiles of three juveniles Rice’s whales (subadult male USNM 239307, female calf 593537, and subadult female 504768) from observed immunoreactive hormone concentrations across the baleen plate. Hormone concentrations (ng/g) are plotted by distance from base (0 being the base, i.e., the point grown most recently before death) to read from oldest (left) to newest (right) baleen growth. Dashed horizontal lines represent baseline and black points represent peaks, defined as values two standard deviations or more above baseline. For display purposes, two progesterone outliers for whale USNM 239307 have been removed (202.56 ng/g at 8 cm and 269.82 ng/g at 5 cm).

## Discussion

Our study confirms that steroid hormones can be accurately quantified in Rice’s whale baleen, with progesterone, testosterone, cortisol and corticosterone all providing useful information regarding reproductive seasonality (and lack thereof), pregnancy and parturition, and indication of chronic stress prior to death.

### Adult males

Our data show no clear evidence of regular annual elevation of testosterone in three adult male Rice’s whales, and thus, no clear evidence of reproductive seasonality. In many other adult male mysticetes, cyclical testosterone patterns in baleen typically occur during certain months of the year that correspond to putative breeding seasons [39,56]. The closely related Bryde’s whale, typically also found in tropical waters, can breed year-round but does have heightened breeding activity in autumn (September to November) [57]. In this study, the three adult male baleen plates capture two autumns, with the first containing no testosterone peaks in any of the three males. The second autumn season, however, does contain testosterone peaks from two of the three adult males (USNM 504074 and USNM 594665). Regardless, additional elevations in testosterone are present across the plate, indicating that testosterone does not only rise during potential breeding seasons but can rise at any point in the year. Overall, these patterns are reminiscent of Bryde’s whales, as well as of tropical and subtropical mammals generally (i.e., it is plausible that Rice’s whales might tend to breed in autumn while also retaining some potential to breed year-round).

Our interpretation of testosterone patterns may be influenced by various study limitations such as low sample size and potential inaccuracies in timeline estimates. Only three male individuals were available for study, with each baleen plate spanning only two years. Further, all estimated dates-of-growth are based on the baleen growth rate of the Bryde’s whale. It is possible that Rice’s whales may have a different baleen growth rate as a species, and additional individual variation may also occur; however, available literature indicates fairly consistent baleen growth rates for adult mysticetes, even across species of similar body size [31,48]. One specimen, USNM 504074, presents the additional complication of potentially missing ~1 cm of the proximal-most baleen; explorations of alternative timelines for this individual indicate that its testosterone peak still falls in autumn. In addition, two of the adult males (USNM 572992 and 594665) had prolonged causes of death, and therefore the peaks of testosterone at the base of the plate might have been affected by physiological status of the whale as it approached death. Overall, our data do not suggest reproductive seasonality in this species, but we recommend further study with any additional baleen specimens that become available.

All three adult males in this study had higher progesterone than testosterone. This may at first seem unexpected, as testosterone is famed as the main reproductive hormone in male mammals, while progesterone is typically studied only in females. However, on the rare occasions when progesterone is studied in males, adult male mammals often do prove to have high progesterone, so much so that it is speculated that progesterone may play a specific role in male reproduction that has not yet been elucidated [58–60]. Alternatively, this pattern may simply reflect the biosynthetic pathway of steroidogenesis, as progesterone is the immediate precursor to testosterone. Indeed, progesterone and testosterone patterns mirror each other in all three adult male whales (i.e., a rise in progesterone corresponds with a rise in testosterone). It is also possible that hormone ratios in baleen may differ from those in plasma (i.e., chemical properties of progesterone and testosterone may impact affinity for binding to keratin and thus the efficacy of embedding into keratin structures). Emerging data indicate this pattern of high progesterone in male mysticetes does occur in baleen of other species such as blue, fin, and bowhead whales (A. Case, J. Jelincic, unpublished data), but few-to-no plasma samples are available from male mysticete whales for comparison. The potential biological significance of high progesterone in male mysticetes therefore remains to be determined.

### Adult female

A prolonged elevation in progesterone, with a gradual rise and then a gradual fall, is evident in the adult female’s baleen. This pattern is consistent with expected progesterone levels during pregnancy. Indeed, this individual was known to be lactating at her point of death and therefore must have been pregnant in the prior year (the year captured by the baleen). Our estimated gestation duration of 13.7 months, though likely imprecise, provides a first look at gestation length in this species based on physiological data, and generally agrees with approximately year-long gestation lengths of the Bryde’s whale and related species [56,61]. Though our sample size is a single individual, this case, along with similar results from other species, strongly suggests that prior-year pregnancies of Rice’s whales can be diagnosed accurately from patterns in baleen progesterone.

The proximal-most portion of this female’s baleen plate contained a near-simultaneous rise in testosterone, cortisol and corticosterone at approximately 1–2 cm before the base of the baleen plate (i.e., approximately two months before death). This cluster of endocrine changes at approximately 2 cm may represent the parturition event and/or onset of lactation, physiological events that both can involve elevations in GCs [62,63]. Testosterone is not often assessed in pregnant female mammals, but some data indicate that androgens from male fetuses may be detectable in maternal circulation, and, further, male fetuses in many mammal species have a notable rise in androgen secretion near or at birth [64,65]. Alternatively, endocrine changes near the base of a whale’s baleen plate can sometimes be attributed to a chronic cause of death (e.g., chronic injury or illness) [19,20]. However, in this case, necropsy and observational (carcass sighted on bow of vessel) data confirm an acute cause of death of vessel strike. We expect a ship strike (i.e., a near-instantaneous death), as experienced by the adult female, would not cause an increase in GCs at the 1–2 cm region of the plate.

### Subadults and calf

The single calf in this study had the highest baseline levels of all four hormones analyzed, including the reproductive hormones (testosterone five times higher than adult baseline, and progesterone almost four times higher than adult baseline). Similarly, the two subadults also had hormone concentrations higher than adults (testosterone and progesterone both three times higher than adult baselines). Because calves are not reproductively active, there may be another role for progesterone and testosterone at this age. Progesterone and testosterone may be of maternal origin, transferred via milk fat during lactation [66–68]. With the one pregnant female included in this study, testosterone was elevated toward the base of the plate when she would have been lactating, potentially explaining high testosterone in calves if it is indeed due to maternal transfer via milk. A second explanation could be that these hormones are endogenously produced in the young whales, perhaps relating to sex determination; for example, neonatal males of several mammalian species produce very high endogenous androgens in the months just after birth, thought to be related to endogenous sex determination that occurs post-partum [69–71]. However, the calf and two subadults all have higher progesterone levels than testosterone regardless of sex. Yet, as seen in adult males, some males have higher progesterone baselines compared to testosterone. Reproductive hormones are rarely reported for neonatal mammals, so it is unclear how widespread these patterns might be or what their physiological purpose may be. We encourage more routine analysis and reporting of reproductive steroids in calf and subadult age classes of all mysticete species.

### Chronic stress events

Three whales in this study have known causes of death. Two involved prolonged stressors; one such case involved plastic ingestion that perforated the gastrointestinal tract numerous times, causing chronic illness and hemorrhaging [72] (USNM 594665), while the other involved chronic entanglement in fishing gear that cut deeply into bones of the skull (USNM 572992). Poor body condition was also reported at necropsy (i.e., starvation). These two whales with prolonged death events had marked increases in all four hormones — both reproductive steroids and both GCs — near the base of the plate starting around 4 cm (~2 months prior to death). The rise in GCs agrees with our predictions that chronic stress events should result in elevated GCs in baleen. The rises in progesterone and testosterone, however, are more puzzling, but similar patterns have been noted in several other cases of chronic cause of death in other mysticetes (e.g., [19]). One explanation for the increased hormones could be adrenal failure and/or gonadal failure, as both of these glands produce a wide suite of steroid hormones via complex biosynthetic pathways in which various hormones are precursors to other hormones, and thus, gradual failure of enzyme production or activity could result in the release or “dumping” of large amounts of the intermediates into plasma. However, a similar pattern of an increase near death has also been reported for contaminants measured in Rice’s whale baleen, e.g., PFAS spiking in concentration near the base of the plate (baleen grown most recently prior to death) [73]. This suggests the alternate explanation that liver and kidney failure in the moribund animal may result in reduced clearance rates of both hormones and ecotoxicants, causing a global increase in plasma concentrations of multiple types of analytes. Reductions in BGR close to death could result in a similar phenomenon. Regardless of mechanism, we propose that a “moribund pattern” exists in mysticete whale baleen, wherein a whale nearing death from a prolonged stressor accumulates unusually high concentrations of all steroid hormones, and potentially ecotoxicants as well, in the proximal-most baleen.

The third whale with a known death event was the lactating female, who died acutely from a ship strike, likely upon impact (USNM 593536). However, she had also experienced a recent parturition event, which is known to increase GCs in female mammals. This individual did have a rise in GCs a few weeks prior to death, which we tentatively attribute to the parturition (see above).

Of the whales with unknown deaths, we see an increase of GCs starting around the 4 cm mark for the adult male USNM 504074, one peak of B at the 0 cm mark for the subadult male USNM 239307, and no GC peaks in subadult female USNM 504768 and female calf USNM 593537 (though the latter two individuals had baseline GCs throughout the baleen). With further study of cases of known cause-of-death in this and other species (as seen in humpback whales, *Megaptera novaeangliae*; North Atlantic right whales; and southern right whales; [21,23]), it may become possible to identify acute vs. chronic causes of death based on GC patterns in baleen.

## Conclusions

Baleen can be used to retrospectively analyze endocrine states in species that may be rare or from which collection of other sample types is challenging. The Rice’s whale is in urgent need of conservation, with its extremely small population, restricted range, and regular exposure to multiple anthropogenic stressors in the northeast GoM. Here, we demonstrate that baleen hormonal analysis may provide information useful for Rice’s whale management and conservation. For example, through quantifications of testosterone in three adult males, we tentatively conclude that there is no fixed breeding season in Rice’s whales. In addition, with our analysis of progesterone, we demonstrate that pregnancy can be diagnosed in this species in baleen through patterns of elevated progesterone. Lastly, our analysis of GCs indicates that GCs are elevated at the base of the plate in individuals with prolonged deaths. In an unexpected finding, subadults and calves had the highest concentrations of all four hormones, even reproductive hormones, when compared to the mature adults. Increasing the sample size would be beneficial in confirming these findings; genetic investigation of specimens originally categorized as Bryde’s whale may reveal additional Rice’s whale specimens. Overall, endocrinological analyses of baleen provides a powerful tool for obtaining life-history information such as maturation, breeding, pregnancy, and cause of death, information that is vital for conservation efforts.

## Supporting information

S1 Table Full hormone concentration data. All subsample concentrations are presented in ng/g for each of the four hormones quantified.
(PDF)

## References

[1] Rosel P, Wilcox L. Genetic evidence reveals a unique lineage of Bryde’s whales in the northern Gulf of Mexico. Endang Species Res. 2014;25(1):19–34. doi: 10.3354/esr00606

[2] Rosel PE, Wilcox LA, Yamada TK, Mullin KD. A new species of baleen whale (Balaenoptera) from the Gulf of Mexico, with a review of its geographic distribution. Mar Mamm Sci. 2021;37:577–610.

[3] Mullin KD. Abundance of cetaceans in the oceanic Northern Gulf of Mexico from 2003 and 2004 ship surveys. Miami: NOAA National Marine Fisheries Service, Southeast Fisheries Science Center. 2007.

[4] Rosel PE, Corkeron P, Engleby L, Epperson D, Mullin KD, Soldevilla MS, et al. Status review of Bryde’s whales (Balaenoptera edeni) in the Gulf of Mexico under the Endangered Species Act: 32.2 MB. Miami: National Oceanic and Atmospheric Administration, National Marine Fisheries Service, Southeast Fisheries Science Center. 2016.

[5] NOAA Fisheries. Rice’s whale core distribution map & GIS Data. NOAA Fisheries. https://www.fisheries.noaa.gov/resource/map/rices-whale-core-distribution-area-map-gis-data 2019.

[6] Reeves RR, Lund JN, Smith TD, Josephson EA. Insights From Whaling Logbooks on Whales, Dolphins, and Whaling in the Gulf of Mexico. goms. 2011;29(1). doi: 10.18785/goms.2901.04

[7] Širović A, Bassett HR, Johnson SC, Wiggins SM, Hildebrand JA. Bryde’s whale calls recorded in the Gulf of Mexico. Mar Mamm Sci. 2014;30:399–409.

[8] Rice AN, Palmer KJ, Tielens JT, Muirhead CA, Clark CW. Potential Bryde’s whale (Balaenoptera edeni) calls recorded in the northern Gulf of Mexico. J Acoust Soc Am. 2014;135(5):3066–76. doi: 10.1121/1.4870057 24926502

[9] Soldevilla M, Hildebrand J, Frasier K, Aichinger Dias L, Martinez A, Mullin K, et al. Spatial distribution and dive behavior of Gulf of Mexico Bryde’s whales: potential risk of vessel strikes and fisheries interactions. Endang Species Res. 2017;32:533–50. doi: 10.3354/esr00834

[10] Soldevilla M, Debich A, Garrison L, Hildebrand J, Wiggins S. Rice’s whales in the northwestern Gulf of Mexico: call variation and occurrence beyond the known core habitat. Endang Species Res. 2022;48:155–74. doi: 10.3354/esr01196

[11] Soldevilla MS, Debich AJ, Pérez‐Carballo I, Jarriel S, Frasier KE, Garrison LP, et al. Rice’s whale occurrence in the western Gulf of Mexico from passive acoustic recordings. Marine Mammal Science. 2024;40:1–8.

[12] NOAA Fisheries, Southeast Regional Office. Rice’s whale recovery planning workshop. NOAA Fisheries, Southeast Regional Office. 2021. https://www.fisheries.noaa.gov/s3/2022-04/RIWH_WorkshopSummary_Oct-Nov2021_FinalDraft_Public-Version_508%20Compliant.pdf

[13] Busch DS, Hayward LS. Stress in a conservation context: A discussion of glucocorticoid actions and how levels change with conservation-relevant variables. Biol Conserv. 2009;142:2844–53.

[14] Bryant J, Wielebnowski N, Gierhahn D, Houchens T, Bellem A, Roberts A, et al. Using non-invasive faecal hormone metabolite monitoring to detect reproductive patterns, seasonality and pregnancy in red river hogs (Potamochoerus porcus). J Zoo Aquar Res. 2016;4:14–21.

[15] Pérez-Ortega B, Hendry AP. A meta-analysis of human disturbance effects on glucocorticoid hormones in free-ranging wild vertebrates. Biol Rev Camb Philos Soc. 2023;98(5):1459–71. doi: 10.1111/brv.12962 37095625

[16] Srivastava T, Hameed J, Kumar V, Segu H, Narayan S, John M, et al. Establishing reproductive seasons for the conservation of the critically endangered Kashmir red deer Cervus Hanglu. Sci Rep. 2025;15(1):4955. doi: 10.1038/s41598-025-89244-1 39930058 PMC11811425

[17] Hunt KE, Stimmelmayr R, George C, Hanns C, Suydam R, Brower H Jr, et al. Baleen hormones: a novel tool for retrospective assessment of stress and reproduction in bowhead whales (Balaena mysticetus). Conserv Physiol. 2014;2(1):cou030. doi: 10.1093/conphys/cou030 27293651 PMC4806734

[18] Hunt KE, Lysiak NS, Robbins J, Moore MJ, Seton RE, Torres L, et al. Multiple steroid and thyroid hormones detected in baleen from eight whale species. Conserv Physiol. 2017;5(1):cox061. doi: 10.1093/conphys/cox061 29230292 PMC5691779

[19] Lysiak NSJ, Trumble SJ, Knowlton AR, Moore MJ. Characterizing the duration and severity of fishing gear entanglement on a North Atlantic right whale (Eubalaena glacialis) using stable isotopes, steroid and thyroid hormones in baleen. Front Mar Sci. 2018;5:168.

[20] Fernández Ajó AA, Hunt KE, Uhart M, Rowntree V, Sironi M, Marón CF, et al. Lifetime glucocorticoid profiles in baleen of right whale calves: potential relationships to chronic stress of repeated wounding by Kelp Gulls. Conserv Physiol. 2018;6(1):coy045. doi: 10.1093/conphys/coy045 30151197 PMC6101610

[21] Fernández Ajó AA, Hunt KE, Giese AC, Sironi M, Uhart M, Rowntree VJ, et al. Retrospective analysis of the lifetime endocrine response of southern right whale calves to gull wounding and harassment: A baleen hormone approach. Gen Comp Endocrinol. 2020;296:113536. doi: 10.1016/j.ygcen.2020.113536 32540491

[22] Rolland R, Graham K, Stimmelmayr R, Suydam R, George J. Chronic stress from fishing gear entanglement is recorded in baleen from a bowhead whale (Balaena mysticetus). Mar Mamm Sci. 2019;35:1625–42.

[23] Lowe CL, Hunt KE, Robbins J, Seton RE, Rogers M, Gabriele CM, et al. Patterns of cortisol and corticosterone concentrations in humpback whale (Megaptera novaeangliae) baleen are associated with different causes of death. Conserv Physiol. 2021;9(1):coab096. doi: 10.1093/conphys/coab096 34987826 PMC8710851

[24] Lowe CL, Hunt KE, Rogers MC, Neilson JL, Robbins J, Gabriele CM, et al. Multi-year progesterone profiles during pregnancy in baleen of humpback whales (Megaptera novaeangliae). Conserv Physiol. 2021;9(1):coab059. doi: 10.1093/conphys/coab059 34745632 PMC8567847

[25] Lowe CL, Hunt KE, Neilson JL, Gabriele CM, Teerlink SS, Buck CL. Reproductive Steroid Hormone Patterns in Baleen of Two Pregnant Humpback Whales (Megaptera novaeangliae). Integr Comp Biol. 2022;62(2):152–63. doi: 10.1093/icb/icac070 35671163

[26] Williamson G. Counting and measuring baleen and ventral grooves of whales. Sci Rep Whales Res Inst. 1973;25:279–92.

[27] Pinto SJD, Shadwick RE. Material and structural properties of fin whale (Balaenoptera physalus) Zwischensubstanz. J Morphol. 2013;274(8):947–55. doi: 10.1002/jmor.20154 23640788

[28] Young S, DeméRé TA, Ekdale EG, Berta A, Zellmer N. Morphometrics and structure of complete baleen racks in gray whales (Eschrichtius robustus) from the Eastern North Pacific Ocean. Anat Rec (Hoboken). 2015;298(4):703–19. doi: 10.1002/ar.23108 25737029

[29] Jensen MM, Saladrigas AH, Goldbogen JA. Comparative Three-Dimensional Morphology of Baleen: Cross-Sectional Profiles and Volume Measurements Using CT Images. Anat Rec (Hoboken). 2017;300(11):1942–52. doi: 10.1002/ar.23648 28971628 PMC5656919

[30] Kessler R. A whale’s life, inscribed in baleen. Science. 2015;350(6266):1300–1. doi: 10.1126/science.350.6266.1300 26659032

[31] Hunt KE, Lysiak NS, Moore MJ, Rolland RM. Longitudinal progesterone profiles in baleen from female North Atlantic right whales (Eubalaena glacialis) match known calving history. Conserv Physiol. 2016;4(1):cow014. doi: 10.1093/conphys/cow014 27293762 PMC4864594

[32] Best P. Distribution and population separation of Bryde’s whale Balaenoptera edeni off southern Africa. Mar Ecol Prog Ser. 2001;220:277–89. doi: 10.3354/meps220277

[33] Dufour JJ, Fahmy MH, Minvielle F. Seasonal changes in breeding activity, testicular size, testosterone concentration and seminal characteristics in rams with long or short breeding season. J Anim Sci. 1984;58(2):416–22. doi: 10.2527/jas1984.582416x 6706874

[34] Lincoln GA, Lincoln CE, McNeilly AS. Seasonal cycles in the blood plasma concentration of FSH, inhibin and testosterone, and testicular size in rams of wild, feral and domesticated breeds of sheep. J Reprod Fertil. 1990;88(2):623–33. doi: 10.1530/jrf.0.0880623 2109070

[35] Minter LJ, DeLiberto TJ. Seasonal variation in serum testosterone, testicular volume, and semen characteristics in the coyote (Canis latrans). Theriogenology. 2008;69(8):946–52. doi: 10.1016/j.theriogenology.2008.01.010 18359065

[36] Jiménez R, Burgos M, Barrionuevo FJ. Circannual Testis Changes in Seasonally Breeding Mammals. Sex Dev. 2015;9(4):205–15. doi: 10.1159/000439039 26375035

[37] Wang Y, Duo H, Li S, Zhang X, Tao H, Fang Y, et al. Functional regulation of testosterone underlying state transition in seasonal spermatogenesis of Plateau Pika (Ochotona curzoniae). J Mammal. 2024;105:1105–16.

[38] Hau M. Regulation of male traits by testosterone: implications for the evolution of vertebrate life histories. Bioessays. 2007;29(2):133–44. doi: 10.1002/bies.20524 17226801

[39] Hunt KE, Lysiak NSJ, Matthews CJD, Lowe C, Fernández Ajó A, Dillon D, et al. Multi-year patterns in testosterone, cortisol and corticosterone in baleen from adult males of three whale species. Conserv Physiol. 2018;6(1):coy049. doi: 10.1093/conphys/coy049 30254748 PMC6148970

[40] Hunt KE, Buck CL, Ferguson SH, Fernández Ajo A, Heide-Jørgensen MP, Matthews CJD. Male Bowhead Whale Reproductive Histories Inferred from Baleen Testosterone and Stable Isotopes. Integr Org Biol. 2022;4(1):obac014. doi: 10.1093/iob/obac014 35617113 PMC9125798

[41] Bentley PJ. Comparative vertebrate endocrinology. Cambridge, UK: Cambridge University Press. 1998.

[42] Pukazhenthi BS, Wildt DE. Which reproductive technologies are most relevant to studying, managing and conserving wildlife?. Reprod Fertil Dev. 2004;16(1–2):33–46. doi: 10.10371/RD03076 14972101

[43] Wingfield JC, Sapolsky RM. Reproduction and resistance to stress: when and how. J Neuroendocrinol. 2003;15(8):711–24. doi: 10.1046/j.1365-2826.2003.01033.x 12834431

[44] Romero M, Wingfield J. Tempests, poxes, predators, and people: stress in wild animals and how they cope. 1st ed. Oxford, UK: Oxford University Press; 2015.

[45] Koren L, Whiteside D, Fahlman S, Ruckstuhl K, Kutz S, Checkley S, et al. Cortisol and corticosterone independence in cortisol-dominant wildlife. Gen Comp Endocrinol. 2012;177(1):113–9. doi: 10.1016/j.ygcen.2012.02.020 22449618

[46] Best PB, Schell DM. Stable isotopes in southern right whale (Eubalaena australis) baleen as indicators of seasonal movements, feeding and growth. Mar Biol. 1996;124:483–94.

[47] Aguilar A, Borrell A. Growth of baleen along the baleen rack is constant in balaenopterid whales. Polar Biol. 2021;44(6):1223–5. doi: 10.1007/s00300-021-02877-6

[48] Werth AJ, Sformo TL, Lysiak NS, Rita D, George JC. Baleen turnover and gut transit in mysticete whales and its environmental implications. Polar Biol. 2020;43(6):707–23. doi: 10.1007/s00300-020-02673-8

[49] Cherney MD, Fisher DC, Auchus RJ, Rountrey AN, Selcer P, Shirley EA, et al. Testosterone histories from tusks reveal woolly mammoth musth episodes. Nature. 2023;617(7961):533–9. doi: 10.1038/s41586-023-06020-9 37138076

[50] Wright C, O’Day P, Alyamani M, Sharifi N, Auchus RJ. Abiraterone acetate treatment lowers 11-oxygenated androgens. Eur J Endocrinol. 2020;182(4):413–21. doi: 10.1530/EJE-19-0905 32045360 PMC7096060

[51] Brown JL, Goodrowe KL, Simmons LG, Armstrong DL, Wildt DE. Evaluation of the pituitary-gonadal response to GnRH, and adrenal status, in the leopard (Panthera pardus japonensis) and tiger (Panthera tigris). J Reprod Fertil. 1988;82(1):227–36. doi: 10.1530/jrf.0.0820227 3123664

[52] Fanson B, Fanson KV. HormLong: An R package for longitudinal data analysis in wildlife endocrinology studies. PeerJ PrePrints. 2015.

[53] Wickham H. ggplot2: Elegant Graphics for Data Analysis. New York: Springer-Verlag. 2016.

[54] Wickham H, François R, Henry L, Müller K, Vaughan D. dplyr: a grammar of data manipulation. 2025.

[55] R Core Team. R: A language and environment for statistical computing. Vienna, Austria: R Foundation for Statistical Computing. 2021.

[56] Otsuki M, Horimoto T, Kobayashi M, Morita Y, Ijiri S, Mitani Y. Testosterone levels in hair of free-ranging male northern fur seals (Callorhinus ursinus) in relation to sampling month, age class and spermatogenesis. Conserv Physiol. 2021;9(1):coab031. doi: 10.1093/conphys/coab031 34026214 PMC8129824

[57] Kato H, Perrin WF. Bryde’s Whale: Balaenoptera edeni. In: Würsig B, Thewissen JGM, Kovacs KM, editors. Encyclopedia of Marine Mammals. 3rd ed. Academic Press. 2018. p. 143–5.

[58] Oettel M, Mukhopadhyay A. Progesterone: the forgotten hormone in men?. Aging Male. 2004;7:236–57. 15669543 10.1080/13685530400004199

[59] Andersen ML, Tufik S. Does male sexual behavior require progesterone?. Brain Res Rev. 2006;51:136–43. 16386800 10.1016/j.brainresrev.2005.10.005

[60] Wagner CK. The many faces of progesterone: a role in adult and developing male brain. Front Neuroendocrinol. 2006;27(3):340–59. doi: 10.1016/j.yfrne.2006.07.003 17014900

[61] Lockyer C. Review of baleen whale (Mysticeti) reproduction and implications for management. 1984.

[62] Weiss G. Endocrinology of parturition. J Clin Endocrinol Metab. 2000;85:4421–5. 11134087 10.1210/jcem.85.12.7074

[63] Benfield RD, Newton ER, Tanner CJ, Heitkemper MM. Cortisol as a biomarker of stress in term human labor: physiological and methodological issues. Biol Res Nurs. 2014;16(1):64–71. doi: 10.1177/1099800412471580 23338011 PMC3904305

[64] Meulenberg PM, Hofman JA. Maternal testosterone and fetal sex. J Steroid Biochem Mol Biol. 1991;39(1):51–4. doi: 10.1016/0960-0760(91)90012-t 2069866

[65] Makieva S, Saunders PTK, Norman JE. Androgens in pregnancy: roles in parturition. Hum Reprod Update. 2014;20(4):542–59. doi: 10.1093/humupd/dmu008 24643344 PMC4063701

[66] Lu M, Xiao H, Li K, Jiang J, Wu K, Li D. Concentrations of estrogen and progesterone in breast milk and their relationship with the mother’s diet. Food Funct. 2017;8(9):3306–10. doi: 10.1039/c7fo00324b 28835965

[67] Petrullo L, Hinde K, Lu A. Steroid hormone concentrations in milk predict sex-specific offspring growth in a nonhuman primate. Am J Hum Biol. 2019;31(6):e23315. doi: 10.1002/ajhb.23315 31468643

[68] Vass RA, Bell EF, Roghair RD, Kiss G, Funke S, Bokor S, et al. Insulin, Testosterone, and Albumin in Term and Preterm Breast Milk, Donor Milk, and Infant Formula. Nutrients. 2023;15(6):1476. doi: 10.3390/nu15061476 36986206 PMC10051190

[69] Picut CA, Ziejewski MK, Stanislaus D. Comparative Aspects of Pre- and Postnatal Development of the Male Reproductive System. Birth Defects Res. 2018;110(3):190–227. doi: 10.1002/bdr2.1133 29063715

[70] Busch AS, Paturlanne JM, Neuhaus N, Wistuba J, Schlatt S, Juul A, et al. Male minipuberty in human and non-human primates: planting the seeds of future fertility. Reproduction. 2023;166(4):R63–72. doi: 10.1530/REP-23-0036 37606226

[71] Serbis A, Kosmeri C, Atzemoglou N, Lampropoulou A, Giaprou LE, Giapros V. Decoding mini-pouberty and its clinical significance: a narrative review. Med Pharmacol. 2025;6:28.

[72] Rosel PE, Corkeron PJ, Soldevilla MS. Balaenoptera ricei. 2022. doi: e.T215823373A208496244

[73] Savoca MS, Robuck AR, Cashman MA, Cantwell MG, Agvent LC, Wiley DN, et al. Whale baleen to monitor per- and polyfluoroalkyl substances (PFAS) in marine environments. Environ Sci Technol Lett. 2024;11(8):862–70. doi: 10.1021/acs.estlett.4c00409 39959431 PMC11824945

