# Supplementary material for: Baleen hormone analyses reveal stress and reproductive life-history of the critically endangered Rice’s whale (Balaenoptera ricei)
Source: PLoS One. 2026 May 13;21(5):e0347749. doi: 10.1371/journal.pone.0347749 (PMC13170835; doi:10.1371/journal.pone.0347749)
Supplement: S1 Table — All subsample concentrations are presented in ng/g for each of the four hormones quantified. (PDF) [file pone.0347749.s001.pdf]

S1 Table. Full hormone concentration data

whale (USNM ID)	sex	age	sample	distance (cm from base)	hormone	concentration (ng/g)
594665	male	adult	1	0	Progesterone	42.04
594665	male	adult	2	1	Progesterone	22.57
594665	male	adult	3	2	Progesterone	19.17
594665	male	adult	4	3.5	Progesterone	22.17
594665	male	adult	5	4.5	Progesterone	21.47
594665	male	adult	6	5.5	Progesterone	16.54
594665	male	adult	7	6.5	Progesterone	17.19
594665	male	adult	8	8	Progesterone	15.44
594665	male	adult	9	9.5	Progesterone	15.89
594665	male	adult	10	10.5	Progesterone	14.35
594665	male	adult	11	11.5	Progesterone	13.74
594665	male	adult	12	13	Progesterone	14.18
594665	male	adult	13	14	Progesterone	13.32
594665	male	adult	14	15	Progesterone	12.77
594665	male	adult	15	16	Progesterone	12.50
594665	male	adult	16	17	Progesterone	12.55
594665	male	adult	17	18	Progesterone	13.07
594665	male	adult	18	19	Progesterone	13.55
594665	male	adult	19	20	Progesterone	14.86
594665	male	adult	20	21	Progesterone	17.96
594665	male	adult	1	0	Testosterone	23.18
594665	male	adult	2	1	Testosterone	14.08
594665	male	adult	3	2	Testosterone	11.27
594665	male	adult	4	3.5	Testosterone	13.39
594665	male	adult	5	4.5	Testosterone	13.65
594665	male	adult	6	5.5	Testosterone	10.55
594665	male	adult	7	6.5	Testosterone	10.37
594665	male	adult	8	8	Testosterone	10.51
594665	male	adult	9	9.5	Testosterone	9.49
594665	male	adult	10	10.5	Testosterone	9.71
594665	male	adult	11	11.5	Testosterone	8.32
594665	male	adult	12	13	Testosterone	7.86
594665	male	adult	13	14	Testosterone	7.61
594665	male	adult	14	15	Testosterone	6.73
594665	male	adult	15	16	Testosterone	6.82

594665	male	adult	16	17	Testosterone	8.48
594665	male	adult	17	18	Testosterone	7.76
594665	male	adult	18	19	Testosterone	8.12
594665	male	adult	19	20	Testosterone	7.42
594665	male	adult	20	21	Testosterone	7.90
594665	male	adult	1	0	Cortisol	3.87
594665	male	adult	2	1	Cortisol	2.48
594665	male	adult	3	2	Cortisol	1.48
594665	male	adult	4	3.5	Cortisol	1.68
594665	male	adult	5	4.5	Cortisol	1.24
594665	male	adult	6	5.5	Cortisol	1.38
594665	male	adult	7	6.5	Cortisol	1.10
594665	male	adult	8	8	Cortisol	1.03
594665	male	adult	9	9.5	Cortisol	1.19
594665	male	adult	10	10.5	Cortisol	1.26
594665	male	adult	11	11.5	Cortisol	1.11
594665	male	adult	12	13	Cortisol	1.17
594665	male	adult	13	14	Cortisol	0.48
594665	male	adult	14	15	Cortisol	0.29
594665	male	adult	15	16	Cortisol	0.69
594665	male	adult	16	17	Cortisol	0.57
594665	male	adult	17	18	Cortisol	0.87
594665	male	adult	18	19	Cortisol	0.82
594665	male	adult	19	20	Cortisol	0.80
594665	male	adult	20	21	Cortisol	1.18
594665	male	adult	1	0	Corticosterone	7.95
594665	male	adult	2	1	Corticosterone	4.40
594665	male	adult	3	2	Corticosterone	3.61
594665	male	adult	4	3.5	Corticosterone	3.78
594665	male	adult	5	4.5	Corticosterone	3.51
594665	male	adult	6	5.5	Corticosterone	2.25
594665	male	adult	7	6.5	Corticosterone	2.23
594665	male	adult	8	8	Corticosterone	1.99
594665	male	adult	9	9.5	Corticosterone	2.20
594665	male	adult	10	10.5	Corticosterone	2.04
594665	male	adult	11	11.5	Corticosterone	1.96
594665	male	adult	12	13	Corticosterone	1.77
594665	male	adult	13	14	Corticosterone	1.58
594665	male	adult	14	15	Corticosterone	1.41

594665	male	adult	15	16	Corticosterone	1.32
594665	male	adult	16	17	Corticosterone	1.09
594665	male	adult	17	18	Corticosterone	1.61
594665	male	adult	18	19	Corticosterone	1.51
594665	male	adult	19	20	Corticosterone	1.87
594665	male	adult	20	21	Corticosterone	2.40
572992	male	adult	1	0	Progesterone	23.31
572992	male	adult	2	1	Progesterone	17.89
572992	male	adult	3	2	Progesterone	17.42
572992	male	adult	4	3	Progesterone	15.80
572992	male	adult	5	4	Progesterone	20.15
572992	male	adult	6	5	Progesterone	11.24
572992	male	adult	7	6	Progesterone	14.96
572992	male	adult	8	7	Progesterone	13.46
572992	male	adult	9	8	Progesterone	13.35
572992	male	adult	10	9	Progesterone	16.06
572992	male	adult	11	10	Progesterone	9.06
572992	male	adult	12	11	Progesterone	14.03
572992	male	adult	13	12	Progesterone	14.72
572992	male	adult	14	13	Progesterone	18.69
572992	male	adult	15	14	Progesterone	19.65
572992	male	adult	16	15	Progesterone	19.63
572992	male	adult	17	16	Progesterone	13.67
572992	male	adult	18	17	Progesterone	14.84
572992	male	adult	19	18	Progesterone	18.79
572992	male	adult	20	19	Progesterone	17.12
572992	male	adult	21	20	Progesterone	22.19
572992	male	adult	22	21	Progesterone	24.20
572992	male	adult	1	0	Testosterone	6.33
572992	male	adult	2	1	Testosterone	3.20
572992	male	adult	3	2	Testosterone	4.30
572992	male	adult	4	3	Testosterone	4.48
572992	male	adult	5	4	Testosterone	4.71
572992	male	adult	6	5	Testosterone	2.64
572992	male	adult	7	6	Testosterone	3.65
572992	male	adult	8	7	Testosterone	3.39
572992	male	adult	9	8	Testosterone	4.58
572992	male	adult	10	9	Testosterone	5.01
572992	male	adult	11	10	Testosterone	3.19

572992	male	adult	12	11	Testosterone	4.22
572992	male	adult	13	12	Testosterone	4.32
572992	male	adult	14	13	Testosterone	5.46
572992	male	adult	15	14	Testosterone	5.70
572992	male	adult	16	15	Testosterone	5.42
572992	male	adult	17	16	Testosterone	5.46
572992	male	adult	18	17	Testosterone	5.81
572992	male	adult	19	18	Testosterone	6.72
572992	male	adult	20	19	Testosterone	6.49
572992	male	adult	21	20	Testosterone	8.42
572992	male	adult	22	21	Testosterone	8.72
572992	male	adult	1	0	Cortisol	2.03
572992	male	adult	2	1	Cortisol	1.19
572992	male	adult	3	2	Cortisol	1.76
572992	male	adult	4	3	Cortisol	1.34
572992	male	adult	5	4	Cortisol	0.08
572992	male	adult	6	5	Cortisol	0.59
572992	male	adult	7	6	Cortisol	0.82
572992	male	adult	8	7	Cortisol	0.60
572992	male	adult	9	8	Cortisol	0.99
572992	male	adult	10	9	Cortisol	0.16
572992	male	adult	11	10	Cortisol	0.59
572992	male	adult	12	11	Cortisol	0.77
572992	male	adult	13	12	Cortisol	0.59
572992	male	adult	14	13	Cortisol	0.40
572992	male	adult	15	14	Cortisol	0.08
572992	male	adult	16	15	Cortisol	0.78
572992	male	adult	17	16	Cortisol	0.08
572992	male	adult	18	17	Cortisol	0.35
572992	male	adult	19	18	Cortisol	0.97
572992	male	adult	20	19	Cortisol	1.00
572992	male	adult	21	20	Cortisol	0.62
572992	male	adult	22	21	Cortisol	1.26
572992	male	adult	1	0	Corticosterone	4.12
572992	male	adult	2	1	Corticosterone	3.30
572992	male	adult	3	2	Corticosterone	2.60
572992	male	adult	4	3	Corticosterone	2.42
572992	male	adult	5	4	Corticosterone	3.96
572992	male	adult	6	5	Corticosterone	1.36

572992	male	adult	7	6	Corticosterone	2.66
572992	male	adult	8	7	Corticosterone	2.03
572992	male	adult	9	8	Corticosterone	1.99
572992	male	adult	10	9	Corticosterone	2.31
572992	male	adult	11	10	Corticosterone	1.39
572992	male	adult	12	11	Corticosterone	1.37
572992	male	adult	13	12	Corticosterone	1.78
572992	male	adult	14	13	Corticosterone	2.16
572992	male	adult	15	14	Corticosterone	2.73
572992	male	adult	16	15	Corticosterone	2.48
572992	male	adult	17	16	Corticosterone	1.78
572992	male	adult	18	17	Corticosterone	1.56
572992	male	adult	19	18	Corticosterone	1.32
572992	male	adult	20	19	Corticosterone	1.52
572992	male	adult	21	20	Corticosterone	3.15
572992	male	adult	22	21	Corticosterone	1.97
504074	male	adult	1	0	Progesterone	42.64
504074	male	adult	2	1	Progesterone	33.67
504074	male	adult	3	2	Progesterone	30.16
504074	male	adult	4	3	Progesterone	48.15
504074	male	adult	5	4	Progesterone	34.71
504074	male	adult	6	5	Progesterone	41.36
504074	male	adult	7	6	Progesterone	39.76
504074	male	adult	8	7.5	Progesterone	46.00
504074	male	adult	9	9	Progesterone	62.32
504074	male	adult	10	10	Progesterone	39.77
504074	male	adult	11	11	Progesterone	36.10
504074	male	adult	12	12	Progesterone	36.05
504074	male	adult	13	13	Progesterone	32.08
504074	male	adult	14	14	Progesterone	32.05
504074	male	adult	15	15	Progesterone	42.90
504074	male	adult	16	16	Progesterone	35.99
504074	male	adult	17	17	Progesterone	28.13
504074	male	adult	18	18	Progesterone	22.87
504074	male	adult	19	19	Progesterone	28.01
504074	male	adult	20	20	Progesterone	29.32
504074	male	adult	21	21	Progesterone	24.75
504074	male	adult	22	22	Progesterone	33.05
504074	male	adult	23	23	Progesterone	43.63

504074	male	adult	1	0	Testosterone	15.46
504074	male	adult	2	1	Testosterone	12.76
504074	male	adult	3	2	Testosterone	14.59
504074	male	adult	4	3	Testosterone	21.45
504074	male	adult	5	4	Testosterone	21.67
504074	male	adult	6	5	Testosterone	16.29
504074	male	adult	7	6	Testosterone	17.49
504074	male	adult	8	7.5	Testosterone	17.43
504074	male	adult	9	9	Testosterone	26.14
504074	male	adult	10	10	Testosterone	18.33
504074	male	adult	11	11	Testosterone	11.03
504074	male	adult	12	12	Testosterone	12.46
504074	male	adult	13	13	Testosterone	10.23
504074	male	adult	14	14	Testosterone	13.56
504074	male	adult	15	15	Testosterone	16.85
504074	male	adult	16	16	Testosterone	12.44
504074	male	adult	17	17	Testosterone	11.76
504074	male	adult	18	18	Testosterone	12.04
504074	male	adult	19	19	Testosterone	12.76
504074	male	adult	20	20	Testosterone	16.33
504074	male	adult	21	21	Testosterone	11.03
504074	male	adult	22	22	Testosterone	17.22
504074	male	adult	23	23	Testosterone	20.57
504074	male	adult	1	0	Cortisol	4.24
504074	male	adult	2	1	Cortisol	4.36
504074	male	adult	3	2	Cortisol	3.60
504074	male	adult	4	3	Cortisol	3.90
504074	male	adult	5	4	Cortisol	3.45
504074	male	adult	6	5	Cortisol	2.40
504074	male	adult	7	6	Cortisol	2.31
504074	male	adult	8	7.5	Cortisol	1.71
504074	male	adult	9	9	Cortisol	2.13
504074	male	adult	10	10	Cortisol	2.16
504074	male	adult	11	11	Cortisol	1.39
504074	male	adult	12	12	Cortisol	0.96
504074	male	adult	13	13	Cortisol	0.55
504074	male	adult	14	14	Cortisol	1.67
504074	male	adult	15	15	Cortisol	1.54
504074	male	adult	16	16	Cortisol	1.34

504074	male	adult	17	17	Cortisol	0.62
504074	male	adult	18	18	Cortisol	1.21
504074	male	adult	19	19	Cortisol	1.14
504074	male	adult	20	20	Cortisol	0.94
504074	male	adult	21	21	Cortisol	0.61
504074	male	adult	22	22	Cortisol	1.14
504074	male	adult	23	23	Cortisol	1.87
504074	male	adult	1	0	Corticosterone	9.42
504074	male	adult	2	1	Corticosterone	7.42
504074	male	adult	3	2	Corticosterone	6.73
504074	male	adult	4	3	Corticosterone	10.09
504074	male	adult	5	4	Corticosterone	5.99
504074	male	adult	6	5	Corticosterone	4.98
504074	male	adult	7	6	Corticosterone	5.66
504074	male	adult	8	7.5	Corticosterone	4.93
504074	male	adult	9	9	Corticosterone	6.06
504074	male	adult	10	10	Corticosterone	4.90
504074	male	adult	11	11	Corticosterone	4.11
504074	male	adult	12	12	Corticosterone	4.73
504074	male	adult	13	13	Corticosterone	3.69
504074	male	adult	14	14	Corticosterone	3.69
504074	male	adult	15	15	Corticosterone	4.12
504074	male	adult	16	16	Corticosterone	3.95
504074	male	adult	17	17	Corticosterone	3.41
504074	male	adult	18	18	Corticosterone	3.34
504074	male	adult	19	19	Corticosterone	3.41
504074	male	adult	20	20	Corticosterone	3.77
504074	male	adult	21	21	Corticosterone	3.05
504074	male	adult	22	22	Corticosterone	3.35
504074	male	adult	23	23	Corticosterone	4.09
239307	male	subadult	1	0	Progesterone	59.66
239307	male	subadult	2	1	Progesterone	48.55
239307	male	subadult	3	2	Progesterone	56.30
239307	male	subadult	4	3	Progesterone	56.08
239307	male	subadult	5	4	Progesterone	269.82
239307	male	subadult	6	5.5	Progesterone	52.76
239307	male	subadult	7	6.5	Progesterone	58.56
239307	male	subadult	8	7.5	Progesterone	42.32
239307	male	subadult	9	8	Progesterone	202.56

239307	male	subadult	10	9	Progesterone	40.06
239307	male	subadult	11	10	Progesterone	30.63
239307	male	subadult	12	11	Progesterone	32.99
239307	male	subadult	13	12	Progesterone	36.85
239307	male	subadult	14	13	Progesterone	35.65
239307	male	subadult	15	14	Progesterone	31.50
239307	male	subadult	16	15	Progesterone	37.88
239307	male	subadult	17	16	Progesterone	44.39
239307	male	subadult	18	17	Progesterone	46.51
239307	male	subadult	1	0	Testosterone	25.26
239307	male	subadult	2	1	Testosterone	21.67
239307	male	subadult	3	2	Testosterone	22.92
239307	male	subadult	4	3	Testosterone	20.60
239307	male	subadult	5	4	Testosterone	33.15
239307	male	subadult	6	5.5	Testosterone	18.37
239307	male	subadult	7	6.5	Testosterone	20.13
239307	male	subadult	8	7.5	Testosterone	19.15
239307	male	subadult	9	8	Testosterone	26.46
239307	male	subadult	10	9	Testosterone	16.14
239307	male	subadult	11	10	Testosterone	12.50
239307	male	subadult	12	11	Testosterone	12.48
239307	male	subadult	13	12	Testosterone	15.97
239307	male	subadult	14	13	Testosterone	14.39
239307	male	subadult	15	14	Testosterone	14.81
239307	male	subadult	16	15	Testosterone	16.96
239307	male	subadult	17	16	Testosterone	21.43
239307	male	subadult	18	17	Testosterone	22.73
239307	male	subadult	1	0	Cortisol	4.49
239307	male	subadult	2	1	Cortisol	4.02
239307	male	subadult	3	2	Cortisol	3.82
239307	male	subadult	4	3	Cortisol	3.38
239307	male	subadult	5	4	Cortisol	9.09
239307	male	subadult	6	5.5	Cortisol	4.15
239307	male	subadult	7	6.5	Cortisol	4.60
239307	male	subadult	8	7.5	Cortisol	3.84
239307	male	subadult	9	8	Cortisol	7.81
239307	male	subadult	10	9	Cortisol	2.77
239307	male	subadult	11	10	Cortisol	2.00
239307	male	subadult	12	11	Cortisol	1.96

239307	male	subadult	13	12	Cortisol	2.22
239307	male	subadult	14	13	Cortisol	2.22
239307	male	subadult	15	14	Cortisol	1.98
239307	male	subadult	16	15	Cortisol	2.17
239307	male	subadult	17	16	Cortisol	2.61
239307	male	subadult	18	17	Cortisol	2.83
239307	male	subadult	1	0	Corticosterone	9.34
239307	male	subadult	2	1	Corticosterone	8.30
239307	male	subadult	3	2	Corticosterone	8.18
239307	male	subadult	4	3	Corticosterone	7.31
239307	male	subadult	5	4	Corticosterone	22.08
239307	male	subadult	6	5.5	Corticosterone	6.08
239307	male	subadult	7	6.5	Corticosterone	5.97
239307	male	subadult	8	7.5	Corticosterone	5.50
239307	male	subadult	9	8	Corticosterone	15.79
239307	male	subadult	10	9	Corticosterone	5.01
239307	male	subadult	11	10	Corticosterone	3.14
239307	male	subadult	12	11	Corticosterone	3.85
239307	male	subadult	13	12	Corticosterone	4.28
239307	male	subadult	14	13	Corticosterone	4.10
239307	male	subadult	15	14	Corticosterone	3.01
239307	male	subadult	16	15	Corticosterone	4.07
239307	male	subadult	17	16	Corticosterone	5.36
239307	male	subadult	18	17	Corticosterone	5.37
593536	female	adult	1	0	Progesterone	462.36
593536	female	adult	2	1.5	Progesterone	575.33
593536	female	adult	3	2	Progesterone	618.14
593536	female	adult	4	3.5	Progesterone	753.00
593536	female	adult	5	5	Progesterone	817.65
593536	female	adult	6	6	Progesterone	913.82
593536	female	adult	7	7.5	Progesterone	939.96
593536	female	adult	8	8	Progesterone	993.39
593536	female	adult	9	9	Progesterone	1181.29
593536	female	adult	10	10	Progesterone	1175.29
593536	female	adult	11	11	Progesterone	1100.63
593536	female	adult	12	12	Progesterone	1020.32
593536	female	adult	13	13	Progesterone	771.17
593536	female	adult	14	14.5	Progesterone	578.20
593536	female	adult	15	16	Progesterone	465.95

593536	female	adult	16	17.5	Progesterone	199.87
593536	female	adult	17	18.5	Progesterone	103.60
593536	female	adult	18	20	Progesterone	91.17
593536	female	adult	19	21	Progesterone	44.78
593536	female	adult	20	22	Progesterone	95.39
593536	female	adult	21	23	Progesterone	167.00
593536	female	adult	1	0	Testosterone	18.84
593536	female	adult	2	1.5	Testosterone	19.59
593536	female	adult	3	2	Testosterone	36.27
593536	female	adult	4	3.5	Testosterone	24.21
593536	female	adult	5	5	Testosterone	14.45
593536	female	adult	6	6	Testosterone	11.76
593536	female	adult	7	7.5	Testosterone	9.81
593536	female	adult	8	8	Testosterone	7.59
593536	female	adult	9	9	Testosterone	8.67
593536	female	adult	10	10	Testosterone	6.25
593536	female	adult	11	11	Testosterone	6.82
593536	female	adult	12	12	Testosterone	5.79
593536	female	adult	13	13	Testosterone	5.89
593536	female	adult	14	14.5	Testosterone	5.60
593536	female	adult	15	16	Testosterone	7.77
593536	female	adult	16	17.5	Testosterone	7.54
593536	female	adult	17	18.5	Testosterone	9.16
593536	female	adult	18	20	Testosterone	11.01
593536	female	adult	19	21	Testosterone	8.20
593536	female	adult	20	22	Testosterone	12.20
593536	female	adult	21	23	Testosterone	11.51
593536	female	adult	1	0	Cortisol	3.50
593536	female	adult	2	1.5	Cortisol	4.62
593536	female	adult	3	2	Cortisol	3.14
593536	female	adult	4	3.5	Cortisol	3.81
593536	female	adult	5	5	Cortisol	1.90
593536	female	adult	6	6	Cortisol	2.03
593536	female	adult	7	7.5	Cortisol	1.26
593536	female	adult	8	8	Cortisol	1.08
593536	female	adult	9	9	Cortisol	1.21
593536	female	adult	10	10	Cortisol	0.88
593536	female	adult	11	11	Cortisol	0.35
593536	female	adult	12	12	Cortisol	1.15

593536	female	adult	13	13	Cortisol	1.01
593536	female	adult	14	14.5	Cortisol	0.81
593536	female	adult	15	16	Cortisol	0.42
593536	female	adult	16	17.5	Cortisol	1.04
593536	female	adult	17	18.5	Cortisol	0.96
593536	female	adult	18	20	Cortisol	0.00
593536	female	adult	19	21	Cortisol	1.08
593536	female	adult	20	22	Cortisol	1.26
593536	female	adult	21	23	Cortisol	0.63
593536	female	adult	1	0	Corticosterone	32.78
593536	female	adult	2	1.5	Corticosterone	41.74
593536	female	adult	3	2	Corticosterone	15.78
593536	female	adult	4	3.5	Corticosterone	11.82
593536	female	adult	5	5	Corticosterone	12.85
593536	female	adult	6	6	Corticosterone	7.79
593536	female	adult	7	7.5	Corticosterone	8.10
593536	female	adult	8	8	Corticosterone	5.89
593536	female	adult	9	9	Corticosterone	6.71
593536	female	adult	10	10	Corticosterone	6.35
593536	female	adult	11	11	Corticosterone	4.46
593536	female	adult	12	12	Corticosterone	5.25
593536	female	adult	13	13	Corticosterone	4.22
593536	female	adult	14	14.5	Corticosterone	3.59
593536	female	adult	15	16	Corticosterone	3.72
593536	female	adult	16	17.5	Corticosterone	2.92
593536	female	adult	17	18.5	Corticosterone	2.94
593536	female	adult	18	20	Corticosterone	3.77
593536	female	adult	19	21	Corticosterone	2.84
593536	female	adult	20	22	Corticosterone	4.47
593536	female	adult	21	23	Corticosterone	3.25
593537	female	calf	1	0	Progesterone	75.19
593537	female	calf	2	1	Progesterone	75.56
593537	female	calf	3	2	Progesterone	59.75
593537	female	calf	4	3	Progesterone	67.11
593537	female	calf	5	4	Progesterone	64.98
593537	female	calf	1	0	Testosterone	43.02
593537	female	calf	2	1	Testosterone	72.63
593537	female	calf	3	2	Testosterone	47.52
593537	female	calf	4	3	Testosterone	44.44

593537	female	calf	5	4	Testosterone	34.39
593537	female	calf	1	0	Cortisol	5.00
593537	female	calf	2	1	Cortisol	7.52
593537	female	calf	3	2	Cortisol	5.44
593537	female	calf	4	3	Cortisol	4.48
593537	female	calf	5	4	Cortisol	4.26
593537	female	calf	1	0	Corticosterone	9.27
593537	female	calf	2	1	Corticosterone	11.48
593537	female	calf	3	2	Corticosterone	7.96
593537	female	calf	4	3	Corticosterone	6.89
593537	female	calf	5	4	Corticosterone	5.53
504768	female	subadult	1	1	Progesterone	35.63
504768	female	subadult	2	2	Progesterone	36.25
504768	female	subadult	3	3	Progesterone	27.80
504768	female	subadult	4	4	Progesterone	24.51
504768	female	subadult	5	5	Progesterone	32.49
504768	female	subadult	6	6	Progesterone	34.49
504768	female	subadult	7	7	Progesterone	42.84
504768	female	subadult	8	8	Progesterone	42.66
504768	female	subadult	9	9	Progesterone	21.87
504768	female	subadult	10	10	Progesterone	40.63
504768	female	subadult	11	11	Progesterone	56.80
504768	female	subadult	12	12	Progesterone	58.06
504768	female	subadult	1	1	Testosterone	29.19
504768	female	subadult	2	2	Testosterone	20.11
504768	female	subadult	3	3	Testosterone	26.88
504768	female	subadult	4	4	Testosterone	19.19
504768	female	subadult	5	5	Testosterone	32.46
504768	female	subadult	6	6	Testosterone	22.95
504768	female	subadult	7	7	Testosterone	19.32
504768	female	subadult	8	8	Testosterone	17.18
504768	female	subadult	9	9	Testosterone	23.95
504768	female	subadult	10	10	Testosterone	22.12
504768	female	subadult	11	11	Testosterone	29.05
504768	female	subadult	12	12	Testosterone	30.82
504768	female	subadult	1	1	Cortisol	4.49
504768	female	subadult	2	2	Cortisol	3.93
504768	female	subadult	3	3	Cortisol	2.61
504768	female	subadult	4	4	Cortisol	3.53

504768	female	subadult	5	5	Cortisol	3.18
504768	female	subadult	6	6	Cortisol	3.36
504768	female	subadult	7	7	Cortisol	2.08
504768	female	subadult	8	8	Cortisol	1.99
504768	female	subadult	9	9	Cortisol	3.05
504768	female	subadult	10	10	Cortisol	2.96
504768	female	subadult	11	11	Cortisol	3.46
504768	female	subadult	12	12	Cortisol	3.21
504768	female	subadult	1	1	Corticosterone	7.20
504768	female	subadult	2	2	Corticosterone	5.78
504768	female	subadult	3	3	Corticosterone	4.80
504768	female	subadult	4	4	Corticosterone	4.35
504768	female	subadult	5	5	Corticosterone	4.51
504768	female	subadult	6	6	Corticosterone	5.20
504768	female	subadult	7	7	Corticosterone	3.49
504768	female	subadult	8	8	Corticosterone	5.39
504768	female	subadult	9	9	Corticosterone	4.86
504768	female	subadult	10	10	Corticosterone	5.91
504768	female	subadult	11	11	Corticosterone	6.51
504768	female	subadult	12	12	Corticosterone	6.86

S1 Table. Full hormone concentration data. All subsample concentrations are presented in ng/g for each of the four hormones quantified.
